# Quality Improvement Guidelines for Transjugular Intrahepatic Portosystemic Shunt (TIPS)

**DOI:** 10.1007/s00270-012-0493-y

**Published:** 2012-10-16

**Authors:** Antonin Krajina, Petr Hulek, Tomas Fejfar, Vlastimil Valek

**Affiliations:** 1 Department of Radiology, University Hospital Hradec Kralove and Medical Faculty of Charles University, 500 05 Hradec Kralove, Czech Republic; 2 Department of Medicine, University Hospital Hradec Kralove and Medical Faculty of Charles University, 500 05 Hradec Kralove, Czech Republic; 3 Department of Radiology, University Hospital Brno and Medical Faculty of Masaryk University Brno, Brno, Czech Republic

## Introduction

In Europe and in North America, portal hypertension accompanies cirrhosis of the liver in over 90 % of the cases. Cirrhosis of the liver is caused by alcohol abuse in about half of the cases; a third is due to chronic viral hepatitis B and C; and the remainder is the result of various metabolic or idiopathic disorders of the liver.

Transjugular intrahepatic portosystemic shunting, as a percutaneous alternative to surgical portosystemic shunts for decompression of symptomatic portal hypertension, was conceived and its technique developed in animal experiments in the late 1960s by Rösch et al. [1].

## Definitions

*Portal hypertension* is a syndrome caused by increased resistance in the portohepatic circulation and an increase in the splanchnic vein blood supply. In the normal liver, the difference between pressures in the portal vein and the free hepatic veins or right atrium usually does not exceed 5 mm Hg. Portal hypertension is defined as a gradient larger than 6 mm Hg, but clinical complications seem to occur only when the pressure gradient exceeds 10–12 mm Hg.

*Wedged hepatic pressure* measurement has two components. The portal component is the pressure transmitted from the hepatic sinusoids, and the systemic component is the blood pressure transmitted from the central veins. It is the portal component that causes the development of portal systemic collaterals. The term *corrected sinusoidal pressure* includes only the portal component and is calculated by subtracting the mean right atrial or inferior vena cava pressure from the wedged hepatic venous pressure. Wedged hepatic pressure is obtained through an end-hole catheter that is advanced into a hepatic vein until it can go no further. Alternatively, pressure can be measured through the wire channel of a double lumen balloon catheter inflated in a more central vein.

*Transjugular intrahepatic portosystemic shunt (TIPS)* is the percutaneous method of creating a portosystemic shunt to decrease or treat portal hypertension. TIPS is a side-to-side shunt of a determined diameter designed to function as a partial shunt that preserves a portion of portal flow to the liver [2]. Flow through the completed shunt is assessed by comparing the degree of preferential filling of the shunt to the that of the portal vein branches and portosystemic collaterals (mainly in the gastric veins). The identification of hepatofugal (reversed) blood flow in portal vein branches (total shunting) is a sign of good flow through the shunt.

*Technical success* is defined as a decrease of the portosystemic pressure gradient to 12 mm Hg or less, or a reduction of at least 20 %.

*Clinical success* is defined as cessation of variceal bleeding, decrease of ascites, and conversion into diuretic-sensitive ascites, as well as improvement of liver function in patients referred for massive thrombosis of hepatic veins.

*Hepatic encephalopathy* is defined as the complex of all cerebral dysfunctions that can occur during the course of serious liver disease. Clinical symptomatology, which as a rule is potentially reversible, ranges from disorientation, somnolence, and lethargy to sopor and coma. Hepatic encephalopathy has three forms: type A, associated with acute liver failure; type B, associated with portosystemic bypass without liver disease; and type C, or chronic, associated with liver cirrhosis.

## Pretreatment Imaging

*Ultrasonography* should verify portal and hepatic vein patency; exclude intrahepatic tumor or cyst; determine maximum blood flow velocities in the portal vein; measure diameters and the congestion index of the main portal, superior mesenteric, and splenic veins; evaluate splenic size; and document the presence and extent of portosystemic collaterals and ascites. This evaluation is the baseline for comparisons to be made during follow-up.

*Computed tomography* (CT) should identify distortion of liver anatomy including lobar atrophy and size of the liver, and evaluate the spatial relationships of the liver and the right kidney, hepatic veins, and portal vein branches. Patency of the portal vein and its tributaries, focal liver lesions, and amount of ascites are also identified by CT.

*Magnetic resonance imaging* measures hepatic flows using a phase contrast technique and provides the most specific imaging diagnosis of the hepatocellular carcinoma using liver cell-specific contrast agent [3].

## Indications for Treatment and Contraindications

### TIPS Indications

Generally, patients with a Child-Pugh score of >12 are in high risk of postprocedural death. A thorough selection of patients is the key to successful treatment with TIPS. Scoring of patients by the model of end-stage liver disease (MELD) has been validated to predict early mortality after TIPS. Patients with MELD scores of >15–18 or a bilirubin level of >60 μmol/L (3.5 mg/dl) should be informed of their poor prognosis, and TIPS should only be performed in the absence of any other treatment possibilities.

#### Refractory Ascites

Refractory ascites is the most frequent indication for TIPS. Refractory ascites is defined as an abdominal fluid collection that cannot be mobilized or the early recurrence of abdominal fluid that cannot adequately be prevented by medical therapy [2]. The term *refractory ascites* has two different meanings: diuretic-resistant ascites and diuretic-intractable ascites. Diuretic-resistant ascites does not respond to an intensified diuretic treatment of up to 400 mg/day spironolactone, up to 160 mg/day of furosemide, and sodium restriction to a maximum intake of 5.2 g of salt/day. Diuretic-intractable ascites cannot be mobilized or their early recurrence cannot be prevented because of the development of diuretic-induced complications that preclude the use of an effective diuretic dosage.

Two important complications occur in patients with cirrhotic ascites: spontaneous bacterial peritonitis and hepatorenal syndrome [4, 5].

Hepatic hydrothorax occurs in patients with ascites when there is direct communication between the peritoneal and pleural cavities. In most patients, the defect is in the diaphragm that overlies the dome of the liver. Hepatic hydrothorax is due to an accumulation of ascitic fluid migrating through the diaphragmatic defect [6].

#### Variceal Bleeding

The causes of gastrointestinal hemorrhage in a patient with portal hypertension may be variceal rupture, portal hypertension gastropathy, postsclerotherapy ulcers, peptic ulcer disease, hemorrhagic gastritis, and Mallory-Weiss tear. TIPS is generally accepted as a second-line therapy after failure of endoscopic and medical therapy of bleeding from gastroesophageal varices.

#### Massive Thrombosis of the Hepatic Veins

TIPS is indicated when medical therapy fails. TIPS is technically feasible in most patients with thrombosis of the hepatic veins. A direct puncture from the intrahepatic inferior vena cava into the liver parenchyma is possible in patients without a remaining hepatic vein stump. The rationale of the use of TIPS is to decompress the liver from venous congestion using the portal vein and TIPS as an outflow tract.

#### Portal Vein Thrombosis

TIPS should be considered in patients with portal vein thrombosis with or without cavernomatous transformation. TIPS can be technically feasible when intrahepatic portal branches are patent. The procedure should be referred to centers with extensive experience [7].

#### Portosystemic Collaterals Embolization

There are three main indications for occluding large portosystemic collaterals. The most frequent is embolization of the left and short gastric veins in a patient with a recent history of acute variceal bleeding, especially if the bleeding source was gastric varices. Another indication is closure of large collaterals with continuing competitive flow after TIPS. In addition, patients with a history of portosystemic encephalopathy or patients at high risk for that condition may benefit from embolization of large portosystemic collaterals performed during calibrated TIPS.

### TIPS Contraindications

TIPS is absolutely contraindicated in cases of unproved portal hypertension (either clinically or anatomically).

TIPS should be carefully considered in the following circumstances: APACHE II score >20, especially in Child C patients, and irreversible phase of hemorrhagic shock; Child-Pugh score >12 and MELD score >18; right-sided heart failure with elevation of the central venous pressure (mean right atrium pressure >15 mm Hg); hepatic encephalopathy poorly controlled by lactulose, especially in patients older than 60, patients with diabetes, and patients receiving hemodialysis; chronic occlusion of the portal vein with periportal collaterals, hypervascular hepatic tumors, polycystic liver disease; and active infection, either intrahepatic or systemic.

To provide TIPS for variceal bleeding, the following laboratory values are required: total bilirubin <60 μmol/L (3.5 mg/dl), especially in Child C patients; and corrected international normalized ratio (INR) of <1.8. To provide TIPS for refractory ascites, the following laboratory values are required: total bilirubin <50 μmol/L (3 mg/dl); and serum creatinine of <180 μmol/L (2.1 mg/dl), except in cases of hepatorenal syndrome. Elevated total serum bilirubin level can also be due to biliary obstruction, hemobilia, hemolysis, or primary biliary cirrhosis. In such cases, values of >60 μmol (3.5 mg/dl) are not a contraindication for TIPS.

The encephalopathy test and the anatomic ultrasound and CT studies can be excluded when a patient is acutely bleeding and cannot undergo endoscopic therapy.

## Patient Preparation

Laboratory tests are performed to reveal coagulopathy, liver and renal failure, and systemic infection, as well as to establish Child-Pugh classification (i.e., prothrombin time/INR, partial thromboplastin time, creatinine, urea, electrolytes, bilirubin, total protein, complete blood count, transaminases). The operator should know at least these values to assess and minimize potential risks arising from iodinated agent administration, structural liver injuries, and overshunting of the portosystemic blood flow [8].

Subclinical hepatic encephalopathy is revealed using a number connecting test. The patient’s signature should be documented in the chart every day.

To improve the patient’s clinical state, hematocrit, protein, coagulation deficit (fresh frozen plasma if INR is >1.8, platelet infusion if count is <50,000 × 10^9^/L, or according to the center’s standards) should be corrected. The acutely bleeding patient has to be hemodynamically stabilized during the procedure.

Cardiologic evaluation should reveal preexisting cardiac dysfunction. Silent cirrhotic cardiomyopathy may be unmasked after TIPS insertion [9].

Prophylactic broad-spectrum antibiotics should be administered. With a potentially limited effect on hepatic metabolism, antibiotics are initiated on the day of procedure and continued for at least two additional days. (Antibiotic therapy should be included in the therapy of variceal bleeding.)

Two cross-matched blood units should be available.

Draining of tense ascites can be performed to decrease the angle between the hepatic veins and the inferior vena cava and have better fluoroscopic imaging. Clinicians should be aware of hepatorenal syndrome. Monitored ascites drainage can improve respiratory comfort during the procedure.

It should be determined whether patients have an allergy to contrast media. In patients with a history of idiosyncratic reaction to iodinated contrast media, a reaction rate of 16–44 % (i.e., 4–8 times more that of the general population) has been reported upon reexposure. Pretreatment with corticosteroids at least 12 h before the administration of contrast medium is recommended to prevent adverse reaction.

Patients should be instructed to restrict their oral intake for at least 6–8 h before the procedure.

Placement of a reliable intravenous line should be possible.

Informed consent—a comprehensive description and an explanation of the reasons for this therapy, including potential consequences—must be provided to the patient or patient’s family. Alternative therapies should be mentioned.

## Equipment Specifications

Monoplane digital subtraction angiography equipment that is capable of performing standard projections, including lateral view, should be available. This equipment should provide high-quality fluoroscopy with zooming and reference imaging. The catheterization laboratory should be equipped with ultrasonography to navigate puncture of the jugular and portal veins. Transcatheter blood pressure measurement should be available during the procedure.

There are two TIPS needle-set designs. The first is a relatively simple set, consisting of a 15- to 16-gauge, 55-cm-long, curved steel needle and teflon catheter. The needle has a sharp, reversed bevel at its distal tip. The puncture is made by advancing the entire device through a liver parenchyma. Correct portal vein access is verified by blood return upon aspiration by a 10 ml syringe filled with contrast medium. The contrast medium is used for immediate opacification of the portal vein (excluding accidental puncture of the hepatic artery or biliary duct). Once the portal vein branch is filled with good distal flow, a 180-cm-long stiff guide wire is introduced through the stiff curved cannula. A modification of this set is available that consists of a blunt outer cannula and sharp inner mandril.

The second type of needle set consists of a 14-gauge metal cannula covered with thin-walled teflon catheter. Inside this long cannula is a needle covered with 5F teflon tapered tip catheter. The cannula secures the wedged position of the set inside the hepatic vein, and its curved tip controls the direction of the puncture. The cannula itself does not cross the parenchyma during each needle pass. Instead, the puncture is made by a forceful push of the 0.038 inch needle toward the portal vein branch while the metal cannula is held in wedged position in the hepatic vein. When this sheathed needle is used, the diameter of the puncture hole is approximately 1.7 mm (i.e., 5F).

Also available is a pediatric TIPS set, which includes a 0.018 inch guide wire instrumentarium.

## Procedure

After entry into the internal jugular vein, a catheter is introduced and guided through the superior vena cava, right atrium, and inferior vena cava into a hepatic vein—usually the right hepatic vein. Wedge hepatic pressure measurement follows. The use of the proximal portion of the hepatic vein has two purposes. The first is to utilize, for shunt creation, the largest diameter of the hepatic vein to potentially prevent or delay any outflow shunt stenosis. The second is to be sure that one begins cephalad to the desired portal vein entry site. A needle inserted through the catheter is then used to puncture the liver from a central portion of the hepatic vein and enter the main portal branch, usually the right portal vein. In the right hepatic vein, the cannula is rotated approximately 90° anteriorly and then advanced and maintained with continual caudal pressure such that it is wedged against the wall of the hepatic vein. When in the middle hepatic vein, the cannula is rotated posteriorly in the some way. Carbon dioxide wedged hepatic venography is used to identify the portal vein [10]. The puncture can be also navigated with ultrasonography. Depending on the anatomy, it might by possible to use a tract from the right hepatic vein to the left portal branch, and vice versa. The needle tract is then dilated by a balloon catheter, establishing a connection between the portal and systemic circulation directly inside the liver parenchyma. The parenchymal tract is kept open by insertion of an expandable metallic stent. A dedicated TIPS stent graft was designed to extend the covered portion to the orifice of the hepatic vein at the inferior vena cava. The only noncovered part of the stent graft, which is 2 cm long, is that part which protrudes into the portal vein. This both anchors the device and allows blood to flow through the interstices of the noncovered portion to the peripheral (parenchymal) portal vein branches.

From the early TIPS experience, the alternative to the dedicated stent graft has been a self-expandable stent used for bridging portal and hepatic veins in a similar way. The bare stents are used for patients at high risk of hepatic encephalopathy or for recanalization of the portal vein. The shunt diameter is finalized by balloon dilatation of the deployed stent graft or stent.

Depending on the diameter of the expandable stent or stent graft used for TIPS creation, various amounts of portal blood are diverted into the systemic circulation, resulting in the decompression of portal hypertension. The size of the balloon catheter is usually 8 mm. Depending on the pressure gradient measured between the portal vein and right atrium after stent or stent graft placement, the larger angioplasty balloon catheter can be used to achieve adequate stepwise decompression.

For patients who are liver transplant candidates, precise positioning of both ends of the stent or stent graft is critical [11].

The needle may exit the liver and lacerate the liver capsule or enter the hepatic artery. Embolization of the parenchymal tract is the first-line treatment to prevent hemoperitoneum.

The TIPS tract must be intraparenchymal, or dilatation of the extrahepatic portion of the portal vein results in fast exsanguination. The frequency of this complication is about 1 %. Entry into the right or left portal vein branch should be at least 1 to 2 cm from the portal vein bifurcation. The direct injection into the dilated tract should be done as soon as possible to reveal potential extravasation. If it is positive, the balloon is again inflated and the stent graft placed to tamponade the extrahepatic leak. According the patient’s blood pressure, fluid volume resuscitation is immediately initiated and the anesthesiologist is called [11].

The final step of the TIPS procedure is placement of pigtail catheter over the portal vein guide wire for follow-up portography and pressure measurement. The post-TIPS blood pressure is measured within the main portal vein. Once the value is stabilized and recorded, the tip of the sheath or pigtail catheter is moved to the hepatic vein or the suprahepatic inferior vena cava, and the blood pressure is again recorded. Thus, at the completion of the TIPS procedure, at least four pressure values will have been obtained: those in the portal vein and hepatic vein (or inferior vena cava) before and after shunt placement.

## Monitoring Patients During TIPS

The TIPS procedure can be performed with conscious sedation in most patients, although children, critically ill or uncooperative patients, and those on artificial ventilation may require general anesthesia. The routine use of general anesthesia is preferred in some centers.

Conscious sedation requires careful patient monitoring and the assistance of a nurse. Midazolam and fentanyl are routinely used with the administration of supplemental oxygen through nasal prongs or a face mask under continuous oxygen saturation monitoring with a pulse oximetry probe and regular blood pressure measurement. These medications are provided in addition to local skin anesthesia to alleviate the considerable pain that accompanies dilatation of the shunt tract. The intensity of pain varies from patient to patient.

Continuous electrocardiogram monitoring is also required, especially while maneuvering the entry guide wire from the right atrium into the inferior vena cava during inadvertent catheter or wire passage into the right ventricle. The contact of catheters or wires with the inner walls of the right atrium or ventricle can cause severe arrhythmias.

## Postprocedural Follow-up

All TIPS patients are followed by serial duplex ultrasonography at 24 h, 1, 3, and 6 months after the TIPS placement, then every 6 months thereafter [12, 13].

An upper endoscopy is scheduled at 6 months in patients treated for gastrointestinal bleeding as a complementary assessment. The patients are continually followed by a hepatologist.

Anticoagulation is not routinely recommended except in patients treated for massive thrombosis of the hepatic veins (Budd-Chiari syndrome). In these cases, strict anticoagulation is mandatory to keep the INR above 2.

Occurrence of hepatic encephalopathy or its worsening is revealed by direct question of the patient, or by obtaining information by asking family members. Specific questions are asked regarding changes in personality, working capacity, sleep behaviors, and power of concentration. There are psychometric tests for the early detection of subclinical encephalopathy, including study of a handwriting specimen, a number-connection test, and a line-tracing test.

When a polytetrafluoroethylene-covered stent graft is used, hepatic encephalopathy may be frequent; unlike bare stents, its occurrence tends to be constant during follow-up. Refractory hepatic encephalopathy can be managed by reducing the shunt diameter via various stenosing devices [14, 15].

## Outcome

Unpredictable shunt patency remains the greatest problem with this method. With conventional bare metal stents, the probabilities of shunt dysfunction after the primary procedure are ~25 and 50 % after 6 and 12 months, respectively. Creation of a TIPS with the use of expanded polytetrafluoroethylene (ePTFE)-covered stent grafts has been reported to reduce the incidence of shunt dysfunction to 13 % at 6 months and 15–20 % at 1 year. The use of ePTFE-covered stent grafts has dealt with the main problem of primary TIPS dysfunction [13]. Similarly, ePTFE-covered stent grafts also have a place in the secondary prevention of TIPS dysfunction and can improve long-term patency [15–19].

Multiple trials and meta-analyses report much better control of refractory ascites with TIPS than with large-volume paracenteses. TIPS may convert diuretic-resistant ascites into diuretic-sensitive ascites. However, survival and transplant-free survival are similar with the two techniques. Encephalopathy occurs more often in patients treated with TIPS than patients with large-volume paracenteses [20, 21]. TIPS results in improvement of renal function, as indicated by a decrease in serum creatinine and blood urea nitrogen levels [22].

TIPS is effective in controlling acute bleeding from varices refractory to medicamentous therapy, and TIPS should be used in preference to surgery. TIPS should be performed only if pharmacologic and endoscopic therapy have failed in the prevention of variceal rebleeding or when rebleeding occurs after sclerotherapy, and in high-risk patients after the first bleeding attack (hepatic venous pressure gradient >20 mm Hg, or Child-Pugh class B and C with active bleeding during endoscopy) [23].

TIPS should be used in the management of patients with massive thrombosis of hepatic veins whose condition fails to improve with anticoagulation. A large retrospective study revealed 1 and 10 year transplant-free survival to be 88 and 69 %, respectively. TIPS patency in this group of patients, who have hypercoagulopathy, was best in those who received a dedicated covered stent [24].

## Complications

The procedure-related morbidity and mortality of TIPS are much lower than for conventional surgical shunts. The reported rate of fatal complications is 1.7 % (range 0.6–4.3 %). This rate has been found to be inversely related to the number of TIPS procedures performed [25]. Fatal periprocedural complications include intraperitoneal hemorrhage as a result of extrahepatic rupture of the portal vein, laceration of the hepatic artery, and transcapsular puncture with the transjugular needle.

Postprocedural complications and adverse effects include impairment of liver function, indicated by an increase in serum bilirubin concentration; occurrence of hepatic encephalopathy; and cardiac volume overload, which might result in deterioration of circulatory function in patients with cirrhosis and with preexisting heart insufficiency.

## Conclusions

Technical and medical advances have been introduced in TIPS practice to improve shunt patency and to increase the effectiveness of hepatic encephalopathy treatment. These advances will expand the indications for TIPS in the future.
